# Editorial: Fusarium Wilt of Banana, a Recurring Threat to Global Banana Production

**DOI:** 10.3389/fpls.2020.628888

**Published:** 2021-01-11

**Authors:** Gert H. J. Kema, André Drenth, Miguel Dita, Kees Jansen, Sietze Vellema, Jetse J. Stoorvogel

**Affiliations:** ^1^Laboratory of Phytopathology, Wageningen University, Wageningen, Netherlands; ^2^Queensland Alliance for Agriculture and Food Innovation, Centre for Horticultural Science, The University of Queensland, Brisbane, QLD, Australia; ^3^Alliance Bioversity International and CIAT, Cali, Colombia; ^4^Rural Sociology Group, Wageningen University, Wageningen, Netherlands; ^5^Knowledge, Technology and Innovation Group, Wageningen University, Wageningen, Netherlands; ^6^Soil Geography and Landscape Group, Wageningen University, Wageningen, Netherlands

**Keywords:** TR4, Panama wilt, disease management, research priorities, pandemic

## The Topic

This Research Topic contains a selection of papers dealing with Fusarium wilt of banana (FWB), also known as Panama disease, that investigate (i) the epidemiology, distribution, infection biology, and diversity of the pathogen, (ii) management practices, and (iii) ways to identify and screen for resistance. The Research Topic arose from the increasing spread and the growing global impact of FWB, affecting a wide range of banana production systems worldwide.

During the inception of this Research Topic an increased understanding of genetic diversity of *Fusarium oxysporum* f.sp. *cubense* (Foc), traditionally considered as the causal agent of FWB, emerged and showed that Foc comprises several different Fusarium species (Ploetz, 2006; Maryani et al., 2019). The so-called Tropical Race 4 (TR4) was found to be genetically distant from other FWB causing species and was described as *Fusarium odoratissimum* (Maryani et al., 2019).

Different strains of this Fusarium species have affected banana production worldwide. Prior to the 1960s, the spread of FWB was primarily caused by so-called Race 1 strains that caused severe losses in the production and export trade in Latin America, which was based almost entirely on the highly susceptible cultivar Gros Michel. The failing management of FWB in Gros Michel eventually convinced the export companies to convert the business to resistant Cavendish cultivars.

TR4 first emerged in Southeast Asia (Ploetz, 1990) and its current rapid spread was analyzed by Ordonez et al. (2015). Subsequent studies showed that the TR4 strain is extremely virulent toward many banana cultivars, including Cavendish cultivars grown in large-scale monoculture plantations for export markets and many banana varieties important for food security and domestic consumption. There are no readily available solutions to manage this disease. Moreover, this global threat connects export trade, strongly dependent on the susceptible Cavendish cultivars, to local production systems wherein a range of banana varieties contributing to food security are also impacted.

This Research Topic aims to provide a platform for information exchange and knowledge sharing. The contributions demonstrate an active research community in search of effective control of FWB. Taken together, the papers provide an overview of our current understanding of the biology and epidemiology of TR4, its management and how integrated and innovative solutions are required and need to be embraced by all stakeholders in an effort to build a sustainable banana industry for the future.

## Background

Bananas evolved in Southeast Asia and are globally the most traded fruit, currently grown throughout the (sub-)tropics. Annual global production of banana and plantain combined is 155 million metric tons (FAOSTAT 2020; data for 2018). Over 400 million people rely on bananas and plantains for food security and for income. Banana and plantains are consumed as fruits or as a starchy food staple and are an important ingredient of local diets. Other uses include; beer brewing, packaging and food wrapping, fibers for clothing and handicrafts, transport pallets and animal feeds and dried sheaths for binding ropes, thatching materials, mulching material and traditional medicines.

Banana production takes place under diverse agro-ecological and social-economic conditions where disease pressure varies significantly. Approximately 84% of the crop is cultivated by smallholders and delivered to domestic markets. The international trade represents about 16% of the global banana production consisting of just over 25 million metric tons which are exported from tropical areas to mainly countries in the temperate zones (FAOSTAT 2020; data for 2018). The latter bananas are almost exclusively of the “Cavendish” variety grown in monoculture in large plantations for export. Cavendish cultivars are also important for domestic markets and represent ~50% of the global banana production. They are resistant to Race 1 strains, but susceptible to TR4. The threat posed by Race 1 was countered by replacing the susceptible Gros Michel with the resistant Cavendish, particularly in export-oriented production systems in tropical lowlands.

Refrigerated transport enabled the export industry to be developed in the 20th century based on the banana variety Gros Michel. This hardy and highly popular variety however turned out to be susceptible to FWB Race 1. After invading Central America Race 1 continued to spread to other banana growing countries and destroyed Gros Michel plantations putting pressure on the export industry in Central and South America (Simmonds, 1966). Despite large scale and expensive efforts to manage FWB in large monoculture plantations, effective control was never achieved. For the first half of the 20th century the industry tried to hold on to “Gros Michel” by shifting cultivation to escape the pathogen with great environmental and socio-economic consequences (Marquardt, 2001; Soluri, 2005). The banana export industry only gradually adopted Cavendish as a replacement for Gros Michel as it required changes to the logistic supply chain. The introduction of cardboard boxes enabled shipment of the more fragile and easily bruised Cavendish fruit, and improved temperature control through refrigeration and artificial ripening, enabled delivery of bananas to Western markets in an acceptable quality. The change in variety as response to Race 1 necessitated major adjustments in logistics and marketing, but the large-scale and uniform production systems based on one single cultivar remained.

Since the 1960's global banana production has expanded significantly due to increased global demand. Cavendish turned out to be highly productive in intensively managed plantations and acceptable to international and domestic markets resulting in contributing approximately 50% of global production and 99% of export markets. The downside of relying on a single cultivar at a global scale is genetic vulnerability which has become evident by the rapid spread of Race 1, and black leaf streak disease, or black Sigatoka (Marin et al., 2003; De Bellaire et al., 2010) in Central and South America and more recently by the emergence and dissemination of TR4. The TR4 epidemic started in the 1960s in Taiwan, and apparently expanded into South East Asia and China and subsequently emerged in the Middle East, Africa, the Indian subcontinent, and most recently in Colombia (García-Bastidas et al., 2020). The spread of TR4 around the world has increased significantly and causes severe damage to the highly susceptible “Cavendish” cultivars planted in huge monocultures as well as backyard gardens.

## The Scope of the Research Topic

The papers in this Research Topic cover a wide range of issues in an effort to capture the biology and epidemiology of the pathogen (Zheng et al.; Dita et al.; Warman and Aitken; Liu et al.; Pegg et al.), the impact and management of the disease (Montiflor et al.; Carvalhais et al.; Bubici et al.; Staver et al., 2020), identification and screening for resistance (Chen et al.; García-Bastidas et al.) and the socioeconomic approaches to engage all stakeholders in coordinated efforts to contain the spread and to exchange knowledge under circumstances of uncertainty and unfamiliarity (Montiflor et al.; Staver et al., 2020).

The export sector represents the more salient part of the worldwide banana sector; however, the consequences of TR4 for banana producers, traders and consumers in local food provisioning systems is also severe. Therefore, the Research Topic aims to connect the two distinct domains, of export trade and local food security (Oosterveer et al., 2014). This multiplicity enables spread of the pathogen and complicates tailoring of disease management to different circumstances. Yet, the truly global nature of TR4 may be conducive to linking the resources and knowledge of the export-oriented industry with the problem-solving capacities and livelihood strategies of banana producers in diverse agro-ecological and socio-economic contexts.

## The Research Papers

### Infection and Spread of the Pathogen

The paper by Pegg et al. deals with the epidemiology of FWB. The paper provides a complete introduction to the FWB problem and this Research Topic. The authors review the body of evidence with regard to the origin, spread and infection of the pathogen and the mechanisms of colonization of the banana plant leading to expression of disease symptoms. The authors outline the evidence for the survival of TR4 in the soil either as chlamydospores or as an asymptomatic endophyte in a wide range of different non-host plants. Issues which prevent effective management of FWB, are discussed in detail. Some crucial points, such as the long incubation period, are highlighted which substantiate the observation that a lack of symptoms may not be a good indication of the presence or absence of the pathogen. In fact, it may be several years before the pathogen present in the soil infect the banana roots and FWB occurrence become evident.

Several fundamental epidemiological issues are brought to the forefront including the long established observation that all infections by Fusarium originate from the roots (Wardlaw, 1961). Banana root exudates stimulate the germination of chlamydospores present in the soil and the initial advancement of the pathogen through the roots is slow but once the pathogen enters the pseudostem it can spread rapidly through the formation of microconidia in the xylem vessels. The authors hypothesize that when Fusarium microconidia in the xylem vessels are confronted with a perforation plate they germinate and the resulting mycelium grows through the perforation plate to form microconidia at the other side, progressing the co-lonization of the pseudostem. In banana varieties resistant to FWB the defenses of the host arrest pathogen colonization in the rootlets, roots or at the root base while in susceptible plants the colonization of the xylem continues unabated. Symptoms expressed as wilting are a result of impaired water movement due to vascular clogging, significantly reducing the transpiration levels. In the final phase of colonization, the pathogen moves from the xylem into the parenchyma and cortex of the plant where an abundance of chlamydospores and conidia are produced in the degrading plant tissue. The paper also gives a pertinent overview of the areas that are insufficiently covered in contemporary research efforts such as; completing the disease cycle, investigating in detail the host pathogen interaction, development and application of effective containment measures, detection of affected plants, destruction of infected plant material, soil treatments to reduce the inoculum load, the process of colonization of the pseudostem and the nature of resistance in Cavendish to Race 1.

Warman and Aitken utilize in their paper a combination of a Subtropical Race 4 strain (SR4) labeled with Green Fluorescent Protein (GFP) and confocal microscopy to visualize pathogenesis in a compatible interaction (Warman and Aitken). Their results show that colonization of the roots and pseudostem is quite advanced before external symptoms of disease become apparent. This GFP study confirms the direct penetration of the epidermal cells by the pathogen in the root tips followed by intercellular growth along the elongation zone and the initial colonization of the xylem vessels in asymptomatic fashion. The mechanisms of the movement of the pathogen throughout the pseudostem remains unresolved. Colonization of the pseudostem is followed by colonization of the leaf sheaths and production of chlamydospores in symptomatic plants. The authors provide some evidence that senescent leaf sheaths may be an important source of inoculum, particularly since decaying leaves also show hypha appearing from stomata on the outside of leaf sheaths, which could result in aerial dissemination of the pathogen and underscores the relevance of leaves in disease epidemiology and management.

The genetic differentiation of Fusarium species causing wilt in bananas is poorly understood and an analysis among Race 1, SR4 and TR4 reveals some interesting findings (Liu et al.). Evidence that R1 has a highly diverse genetic background while TR4 shows a monophyletic origin which was identified previously (Ordonez et al., 2015) is corroborated in this study. The evolutionary characteristics of 12 Fusaric Acid Biosynthesis (FUB) genes and three common household genes are analyzed. The authors find a significantly higher level of recombination among the FUB genes compared to the three common household genes which led them to suggest that horizontal gene transfer plays a role in the evolution of *Fusarium* species causing wilt in bananas confirming previous reports (Ma et al., 2013; Mehrabi et al., 2017; Czislowski et al., 2018). FUB genes are postulated by the authors to be functionally relevant and the presence of negative selection on these genes provides circumstantial evidence that Fusaric acid may play a role as a virulence factor associated with symptoms expression. However, thus far sexuality in Fusarium species causing wilt in bananas is hitherto unknown, and hence, alternative hypotheses need to be tested for the occurrence of recombination in this pathogen (Drenth et al., 2019) and mechanisms of horizontal gene transfer need to be elucidated to gain an understanding of the evolution of new strains and/or species (Zheng et al.).

### Containment, Control, and Coordination

The important question of how much yield loss is avoided by preventing the entry and delaying the spread of the TR4 is addressed by Staver et al. (2020). They show that resources allocated to reducing the spread of TR4 make a very good investment and back-up earlier findings which enumerated the exclusion benefits of keeping TR4 out of Australia (Cook et al., 2015). The authors specifically highlight the danger of touted, but yet unproven, successes such as the use of somaclonal variants and genetic solutions through gene-editing. They argue that such claims may even be a contributing factor to further spread of the disease as they may put the investment in exclusion and containment at risk. The paper clearly shows that investment aimed at reducing the impact of TR4 provides highly positive results. Different scenarios such as improved exclusion, integrated crop and disease management, breeding resistant cultivars and developing genetically modified resistant banana cultivars all significantly reduce future yield losses and provide a high internal rate of return on investment. The paper provides compelling evidence for increased research concerning the management of TR4 in bananas.

Effective implementation of quarantine and containment measures depends on early detection of the pathogen through reliable diagnostics. In addition to morphological identification and vegetative compatibility analyses several molecular diagnostic assays are routinely used to identify TR4. However, since there are multiple races and species of Fusarium causing wilt in a wide range of banana varieties producing similar disease symptoms, a high level of specificity of these tests is paramount. Evidently, one cannot differentiate a Race 1 infection from a TR4 infection in a banana variety susceptible to both and one should not rely on visual symptoms only. To overcome this dilemma Carvalhais et al. developed a molecular diagnostic assay based on Secreted in Xylem (SIX) genes which can reliable detect FWB strains and species, and is able to reliable identify a Race 1, Subtropical (SR4) or TR4 strain. Another important aspect of this paper is the rigor in which the validation was conducted to develop a specific, sensitive and robust diagnostic assay. Reliable detection of different races and species causing FWB enables informed decision making and suitable control measures by banana industry stakeholders in case of an invasion.

Expansion of the geographic distribution of TR4 has been rapid and the disease has spread across Southeast Asia, the Middle East, Africa and more recently to South America (García-Bastidas et al., 2020). Although the pandemic of TR4 is caused by a single clone there are small differences appearing among strains of this clone due to mutations. In a detailed analysis using DNA sequencing to identify single nucleotide polymorphisms (SNP) among TR4 strains, the occurrence of slightly different lineages was demonstrated. Zheng et al. provide a detailed insight into the phylogeography of TR4 by comparing strains collected from various invasions around the world. Using this technology the authors demonstrate that strains from recent incursions in Vietnam, Laos, and Myanmar are genetically similar to the one from Yunnan, China. They also show similarity of strains between the Philippines and Pakistan and a close link between the strains in Lebanon and Jordan. This study shows that genomic analyses of the pathogen can identify individual strains which then can be used to shed light on the origin and distribution of TR4 around the world.

The global distribution of R1 and TR4 and the importance of research on surveillance, exclusion and containment to slow down and limit the spread of TR4 is emphasized in the review of Dita et al. It highlights that research on cropping systems is needed to increase the durability of new resistant clones. The authors outline that Race 1 susceptible varieties such as “Prata” and “Gros Michel,” still can be profitably grown in some regions if combined with rigorous cultural practices. To do this, cultural practices need to significantly reduce the inoculum load in the soil. Hence, information on pathogen survival in the soil and the infection process becomes highly relevant as previously outlined (Warman and Aitken; Pegg et al.). Soil management, including both biotic and abiotic factors, to control FWB is discussed in detail. Factors affecting symptom expression such as weather, temperature, developmental stage of the plant and aspects of different soil components are outlined. However, despite considering all the cultural practices available to us today the authors conclude that control of FWB has not been sufficiently achieved in large scale monoculture systems based on Gros Michel or Cavendish using practical biological, chemical or cultural methods. It is clear that host resistance to FWB has an important contribution to make in sustainable cultivation of bananas.

### Resistance to TR4

Although it is now well understood that “Cavendish” cultivars are susceptible to TR4, it has also become apparent that resistance occurs in other *Musa* germplasm. The paper by Chen et al. describes resistance to TR4 in the diploid AA banana subspecies *Musa acuminata* spp. *malaccensis* and *M. acuminata* spp. *burmannica*. Some polyploid FHIA hybrids, such as FHIA-18 and FHIA-25 are reported to be highly resistant to TR4. Their data reveals that there are different resistance mechanisms present across the 34 *Musa* cultivars they tested and that the resistance response also depends on the inoculum load. They furthermore observe that the resistance response mainly takes place in the rhizome and the outcome of this interaction plays an important role in inhibiting the fungus from colonizing the rest of the banana plant.

The success of plant breeding is largely defined by our ability to select for traits of interest. Therefore, the paper by García-Bastides et al. is fundamentally important as it describes the development of a standard operational procedure to screen germplasm for resistance to TR4, including an inoculum production protocol and additional methodologies underpinning a “high throughput phenotyping system.” In addition, the paper describes how visual scoring is complemented with more objective data capture involving image analysis, contributing to overall reproducibility. A glasshouse-based screening method under controlled conditions using specific pathogen genotypes enables reproducible results in a short period of time, thereby speeding up progress in breeding (Smith et al., 2008; Rebouças et al., 2018).

### Biological Control

In the absence of a market acceptable banana variety with a high level of resistance to TR4 it is not surprising that many efforts have been directed toward finding an effective biological control agent. An overview of articles investigating the prospects of biological control is contributed by Bubici et al. Although there are many studies showing promising leads with efficacy under controlled environmental conditions, none have been taken to the next step to achieve effective control of TR4 in the field. The paper outlines many of the knowledge gaps which exists in this area and the authors make a point that biocontrol should not be considered as an independent tool, but rather as a module of an integrated management framework. Indeed this links back to the paper by Dita et al. who state that a number of integrated cultural practices are required to significantly reduce the inoculum load in the soil to allow Cavendish production in TR4 infested soils.

### Human Factor

Efforts aimed at reducing the impact of any disease are not merely technical but also have substantial social dimensions. The paper by Montiflor et al. looks at the TR4 problem through a different lens and underscores the need to involve all stakeholders in order to contain the disease. The authors argue that efforts should move beyond technical issues and look into how all industry stakeholders in the periphery of the growers, such as industry organizations, researchers, extension workers, exporters and traders, can be involved to partake in the solution (Vellema and Jansen, 2018). The authors investigate issues related to decision making, shared responsibilities and look at how tasks are coordinated and shared at local levels, thereby highlighting the importance of cross-sector collaboration in responding to disease outbreaks and the development of effective partnerships to mobilize local action.

## In Conclusion

The emerging theme of this Research Topic is the reminiscence of the decline of “Gros Michel” due to FWB Race 1 and the current threat of TR4 to global banana cultivation overly dependent on Cavendish cultivars. TR4 affects Cavendish cultivars grown in large scale export-oriented plantations and confronts major banana companies with a recurrent disease problem. However, TR4 also impacts food security and local trade because it affects many other common banana varieties grown for domestic markets. Quarantine and containment may have reduced the spread of TR4 in some regions at a local scale but did not prevent international nor intercontinental dissemination (Drenth and Kema, in press). The application of cultural and biological control options can slow down the development of epidemics, but alone do not provide effective control, thereby merely contributing to gain time. The only long-term option proposed in several papers in this Research Topic is to deploy new varieties with effective disease resistance. This option quenched the Race 1 epidemic once Cavendish replaced Gros Michel. Hence, finding productive and consumer accepted varieties to substitute Cavendish is a key component of strategies to enhance resilience of the export-oriented banana industry as well as many local and regional production systems aimed at domestic markets. Research on genetics, breeding and selection for disease resistance should be accompanied by diversification of the genetic foundations of the banana industry and be combined with other types of solutions, such as soil management or integrating banana with other crops. Changes in the banana industry to control FWD require the involvement of local and international industry stakeholders and consumers to reduce the current extreme level of genetic vulnerability and avoid making the same mistakes once more.

## Author Contributions

All authors listed have made a substantial, direct and intellectual contribution to the work, and approved it for publication.

## Conflict of Interest

The authors declare that the research was conducted in the absence of any commercial or financial relationships that could be construed as a potential conflict of interest.
